# Serum-antibody Profiling of H3N2-infected Ferrets Using a Combinatorial Phage-display Random Peptide Library

**DOI:** 10.1016/j.jmb.2026.169816

**Published:** 2026-04-20

**Authors:** Tehila Yehudai, Gaik Tamazian, Lakshminarasaiah Uppalapati, Sandra Völs, Saranya Sridhar, Guadalupe Cortés, Thorsten U. Vogel, Anna Roitburd-Berman, Jonathan M. Gershoni

**Affiliations:** 1- The Shmunis School of Biomedicine and Cancer Research, George S. Wise Faculty of Life Sciences, Tel Aviv University, Tel Aviv, Israel; 2- Computational Discovery, Compugen Ltd., Israel; 3- Sanofi Vaccines R&D, London, UK; 4- Sanofi Vaccines R&D, Cambridge, MA, USA

**Keywords:** Deep-Panning, machine learning, phage display, IgOme, high-throughput sequencing

## Abstract

The repertoire of antibodies in serum, known as the “IgOme”, is highly diverse and unique to each individual as it reflects the cumulative history of personal encounters with pathogens. Consequently, profiling this repertoire may serve as a diagnostic tool for human viral infections. To explore this potential, we previously developed a computational pipeline called *Motifier*. The pipeline relies on random peptide sequences affinity-selected by monoclonal antibodies, demonstrating that the specifically amplified peptides can act as markers for the antibodies they bind. In this study, we evaluated whether *Motifier* is applicable to highly complex biological samples such as serum, which contain vast collections of antibodies, and whether biological conditions can be identified through serum-profiling. As a model system, we analyzed sera from ferrets infected with H3N2 influenza A strains. Our analyses revealed two principal findings: (i) each ferret displayed a strong and distinct antibody signature, highlighting the dominance of baseline “personal” repertoires; and (ii) peptide–motif markers associated with infection could be identified. Using these infection-related markers, we built a Random Forest classifier, which demonstrated that the markers not only characterized the biological condition but also enabled accurate prediction of unseen samples.

## Introduction

Over the years, phage display [1,2] of random peptides has become a cornerstone tool for studying protein–protein interactions, particularly in immunology [3–11]. Affinity-selected peptides have been used to characterize the epitopes recognized by antibodies. Various methods have been developed where both linear and cysteine-looped random peptide libraries have been employed to reveal the structure and composition of the antigens’ surfaces that interact with their corresponding antibodies, a strategy that has proven useful in epitope mapping [12–22]. A second application of phage display examines the complementary aspect of the antibody–antigen pair, focusing on and characterizing the antibody itself. Identifying and profiling the collection of peptides that bind to a given antibody provide a detailed molecular “signature” that reflects the binding capacity of that antibody. The selected peptides can be epitope relevant and alsoparatope-defining[23,24].

In earlier work, we developed a computational pipeline coined *Motifier* [25] to extract antibody signatures from phage display data combined with high-throughput sequencing (HTS). This process is referred to as Deep Panning (DP) [26]. By applying sequence clustering and alignment algorithms to peptides from a DP experiment, Motifier identifies conserved sequence patterns – motifs – that reflect the binding preferences of antibodies. This method was validated using four monoclonal antibodies (mAbs), each associated with a distinct set of motifs. These motifs, represented as Position-Probability Matrices (PPMs), provided reliable biomarkers of antibody identity and specificity.

Most real-world diagnostic applications, however, examine antibody mixtures such as serum. The antibody repertoire in serum, the “IgOme” [26,27], reflects an individual’s immunological history, including past infections, vaccinations, and other exposures. Unlike mAbs, which are clonally uniform and epitope-specific, polyclonal serum contains a vast diversity of antibodies, including sets of condition-specific antibodies (e.g., those raised in response to a recent infection) mixed among a massive collection of other unrelated antibodies. This complexity presents a major analytical challenge, as the meaningful immunological signals must be extracted from a noisy and highly individualized background [27].

In this study, we apply the Motifier pipeline to the analysis of polyclonal serum, asking whether it can be used to classify serum samples based on a given biological condition, specifically, infection status. The central question was: Can computational motif-based profiling of DP data distinguish between sera from infected *versus* non-infected individuals?

To investigate this, we employed a controlled animal model using Fitch ferrets, divided into two groups: one consisting of non-infected ferrets and the other comprising ferrets infected with one of several H3N2 influenza A strains. Serum samples were collected from both groups and screened against a combinatorial phage-display random peptide library. Affinity-enriched phages were then sequenced using HTS, yielding hundreds of millions of reads representing peptides selected by screening against serum.

The resulting data were analyzed using an updated implementation of Motifier, redesigned for high-throughput performance, modularity and integration with workflow management systems. A major analytical challenge that emerged in the analysis of serum-DP data was the dominance of individual-specific (“personal”) antibody motifs. These motifs occurred in both infected and non-infected ferrets and often drove sample clustering by individual identity rather than infection status. Such personalized signals masked the shared immunological features associated with H3N2 infection.

In conclusion, this study aimed to determine whether peptides derived from DP of polyclonal serum can define robust molecular signatures for immune classification. By extending phage display analysis from monoclonal to polyclonal contexts, we sought to identify condition-associated patterns within the complex landscape of the antibody repertoire.

## Materials and Methods

### The TAU combinatorial phage-display random peptide library (TAU library)

The combinatorial phage-display random peptide library used in this study was produced in-house at Tel Aviv University using the fth1 88 filamentous fd bacteriophage system, which includes an additional, recombinant *protein VIII* gene [28]. The library is comprised of >10^10^ random peptides, either linear or cysteine-constrained looped, ranging from 6 to 12 amino acids in length. For a detailed description of the library construction, characterization, and use, refer to Ryvkin et al. [29]. Generally, to optimize peptide randomness and avoid stop-codons, the library was constructed using preferred phosporamidites ratios for both the first two randomized nucleotides (N) and the third nucleotide (K), which was restricted to G or T (The Midland Certified Reagent Company, Inc) and produced in *SupE*-positive bacteria [30,29]. Repeated HTS analyses of the optimized library revealed a collection of some 700 “irrelevant” peptides that are sometimes enriched post-processing across the variety of biological samples tested. These peptides, considered “parasitic” [31] contaminants unrelated to the biological condition under study, were excluded *in silico* from downstream analyses. The complete list of excluded peptides is provided as Supplementary File S1 (FASTA format).

### Serum samples

Serum samples were obtained through a collaboration with Sanofi Vaccines. All animal procedures were conducted in accordance with the Public Health Service Policy on Humane Care and Use of Laboratory Animals [32] and the Guide for the Care and Use of Laboratory Animals [33], under protocols approved by the Sanofi Institutional Animal Care and Use Committee. Outbred male and female Fitch ferrets (*Mustela furo*, 16–18 weeks old, ≥ 500 g, seronegative by HAI for current seasonal influenza vaccine strains, Marshalls Farms, North Rose, NY) were housed under specified pathogen-free conditions and acclimatized for 7 days before study initiation.

Ferrets were randomized into experimental groups (2–3 animals per group) and intranasally challenged on day 0 with reassortant H3N2 influenza viruses generated by reverse genetics [34]. Each reassortant expressed the targeted Hemagglutinin 3 (HA3) antigen, a neuraminidase (NA) derived from A/HongKong/4801/2014 (H3N2), and internal genes from A/Puerto Rico/8/1934 (H1N1). Non-infected control - “challenged” ferrets received PBS (1000 lL/dose, split in half between nostrils). Blood samples used in this study were collected under sedation at day 21 post-challenge. Sera were isolated and stored at −20 °C until use.

The distribution of animals across groups and infection conditions are provided in Table A. In view of the fact that the training set and the test set were carefully selected and have no ferret overlap or shared serum samples, the potential problem of data leakage was avoided.

### Deep Panning

The DP procedure was essentially as previously described [26,29] and implemented as a unified workflow combining iterative phage display-based selection with HTS as the downstream readout. Briefly, approximately 1 × 10^10^ phages of the TAU combinatorial phage-display random peptide library were mixed with ~10 μg of serum in TBS/3% BSA solution (Tris Buffered Saline – 50 mM Tris–HCl pH 7.5, 150 mM NaCl containing 3% BSA) completed to 100 μl in 0.5 ml vials (AXYGEN, PCR05-C) and incubated for 1 h at room temperature on a rotating mixer. Next, 50 ml of protein G-coated, magnetic beads (DynabeadsTM Protein G, Invitrogen) were added to the mix and incubated for an additional 30 min at room temperature on a rotating mixer. The vials were then placed on a magnetic stand (Promega, MagneSphere^®^ Technology Magnetic Separation Stands) for 2 min to collect the beads and the supernatants were discarded. The beads were washed three times with 200 ml TBS containing 0.5% Tween20 (TBS/T), resuspended in 100 ml TBS/T, and transferred to a new vial. The vials were then placed on the magnetic stand to collect the beads and the supernatants were discarded. The bound phages were eluted twice with 105 μl of elution buffer (100 ml of 0.1 N HCl adjusted to pH 2.2 with 0.2 M glycine, and then 1 mg/ml BSA was added), while rotating at room temperature for 10 min and neutralized with 19 μl of neutralizing buffer (1 M Tris–HCl, adjusted to pH 9.1). Two additional rounds of phage amplification and screening were carried out for each serum sample. Finally, the DNA of phages eluted from the third round of screening was amplified by a Polymerase Chain Reaction (PCR) using the Platinum^™^ SuperFi^™^ Green PCR Master Mix (Invitrogen) and forward (AATGATACGGCGACCACCGAGATCTACACTCTTTCCCTACACGACGCTCTTCCGATCTNNNNNNNNAGGCGGCCAACGTGGC) and reverse (CAAGCAGAAGACGGCAT ACGAGCTCTTCCGATCTGGTCGGCCCCAGCGGC) primers. The thermal profile of the PCR was:(1) 94 °C, 5 min; (2) 94 °C, 30 sec; (3) 60 °C, 30 sec; (4) 72 °C, 30 sec; (5) repeat steps 2–4 for 25 cycles; (6) 72 °C, 5 min. Adapters A and B for Illumina sequencing and eight-nucleotide sample index-barcodes to allow multiplexing (underlined NNNNNNNN in the forward primer) were introduced during PCR [29]. The PCR products were validated for size by running in 2% agarose gels and purified using Agencourt AMPure XP PCR Purification kit (Beckman Coulter, A63881). The concentration of the purified PCR products was measured using a Qubit 2.0 fluorometer (Life Technologies, Q32866), diluted to 5–10 nM and sent for Illumina sequencing at the Weizmann Crown Institute for Genomics using the NextSeq 500/550 High-Output Kit v2.5 (75 Cycles, Illumina).

Each serum sample was processed in three independent replicates. Each replicate was amplified in a separate PCR reaction, generating a distinct PCR product. The resulting PCR products were subjected to single-end Illumina sequencing, producing 84-bp reads that were subsequently processed into FASTQ files for downstream analysis.

### Motifier pipeline

The *Motifier* pipeline is a computational framework designed for IgOme profiling using HTS data derived from DP combinatorial phage-display random peptide libraries. The pipeline comprises three main modules: (i) Quality control;(ii) Motif inference; and (iii) Visualization and machine-learning classification.

The core algorithm and computational pipeline employed in this study are based on the *Motifier* framework. For a detailed description of the original algorithm, including peptide clustering, motif inference, and PPM generation, see the original publication [25].

Since its initial release, the *Motifier* protocol has been applied to multiple DP phage-display experiments. Based on practical experience with the original code, we identified the need for a more efficient and streamlined implementation. Consequently, we re-implemented the pipeline to support efficient and reproducible analysis of peptide sequencing data. Key improvements include optimized runtime, enhanced visualization outputs, and the addition of an integrated prediction module for evaluating classifier performance on unseen data.

The re-implementation was developed in Python 3.13 and C++23 as a set of portable Python packages, with sub-commands for each processing step, managed using Poetry (version 1.8.4) [35]. Workflow execution was orchestrated via Snakemake (version 8.27.1) [36]. The code and workflow files are available at https://github.-com/tehilayehudai/motifier-experiments.

Several parameter values were adjusted from the original publication to align with updated experimental findings and computational requirements.

#### (I) Module 1 – Quality control

Peptides were filtered according to criteria similar to those described in the original publication. Specifically, peptides with lengths ranging from 4 to 12 amino acids, with or without flanking cysteines, were retained. The remaining sequences were normalized to reads-per-million (RPM) to account for varying sequencing depths across samples.

A detailed summary of this stage is provided in Supplementary Table S2.

#### (ii) Module 2 – Motif inference of a biological condition

Peptides generated for each sample served as input for motif inference. For instance, to infer motifs for the” infected” condition in the training set, we analyzed 9 infected ferrets, each screened independently three times against the TAU library, resulting in 27 samples (barcodes).

Motif inference was conducted in two steps: first, motifs were defined for each individual sample (barcode), and second, similar motifs across samples within the same condition were merged.

The algorithms employed were consistent with the original publication, with updated parameters: clustering of peptides was performed using CD-HIT (version 4.8.1) with a 60% sequence identity cutoff, word length = 2, and most similar cluster algorithm mode (mode = 1) [37,38]. For each peptide cluster, multiple sequence alignment was generated using MAFFT (version 7.525) with the ‘auto’ mode and default settings [39,40], producing a PPM.

PPMs were compared by aligning their consensus sequences globally using the Needleman-Wunsch algorithm [41], applying an alignment score threshold of 24. The Pearson Correlation Coefficient (PCC) [42–44] between aligned PPMs was calculated, excluding gapped positions. PPMs with PCC values exceeding 0.7 were unified.

Finally, the top M = 400 most abundant motifs were selected for subsequent analyses.

Assignment of peptides to specific motifs was performed as described in the original publication, without modifications. Assigned peptides were used to produce the Hits table.

#### (iii) Module 3 – Visualization and machine-learning classification

### Clustered heatmap

The Hits table, which consisted of M motifs, was log 2 normalized and visualized using a clustered heatmap (henceforth referred to as a “clustermap”) using the seaborn package (version 0.13.2) [45]. Clustering was performed using hierarchical clustering with the Euclidean distance to calculate the pairwise distances between the samples (rows) and/or the motifs (columns), and the Unweighted Pair Group Method with Arithmetic Mean (UPGMA) algorithm to determine how clusters were merged. Both as implemented in the SciPy package (version 1.14.1) [46]. The dendrogram on the left illustrates the similarity between the samples, and the dendrogram above shows the similarity in motif patterns across samples.

To facilitate reading the clustermap figures, we added a label tag to the clustermap to indicate the biological condition from which each sample originated. By default, samples from the biological condition of interest (i.e., the biological condition from which the motifs were derived) are shown in pink, while samples from the other biological condition (referred to as “Other”) are shown in gray.

### Random forest machine-learning classification

We adopted the random forest (RF) classification framework described in the original *Motifier* publication, with the following adjustments. A 2-fold stratified cross-validation [47] scheme was used. Feature selection was not performed iteratively by reducing the feature set; rather, models were evaluated using the full set of features. Additionally, we sampled 100 hyperparameter configurations from a predefined grid, instead of 1000. All models were implemented using the Scikit-learn library (version 1.5.2) [48] and feature importance was assessed using the Gini impurity [49,50] criterion.

### Prediction

This process involved repeating the step of associating peptides to motifs, as defined in the training set, but now applied to the test set samples. A new Hits table was generated, with columns representing the motifs defined from the biological condition of interest in the training set and rows representing the test samples. The test set included samples from both the biological condition of interest and the “Other” biological condition (infected and non-infected in our experiment).

The resulting Hits table, along with the trained model, was used for prediction to determine whether test samples were correctly classified according to their original biological condition.

### Confusion matrix

To evaluate prediction results on unseen samples and assess whether the predicted labels matched true labels, we calculated a confusion matrix using the metrics module from Scikit-learn (version 1.5.2) [48]. The matrix was visualized as a heatmap using the seaborn package (version 0.13.2) [45].

### Sample visualization

To visualize the distribution of samples across motifs in a lower-dimensional space, we applied Uniform Manifold Approximation and Projection (UMAP), a non-linear dimensionality reduction technique implemented via umap-learn package (version 0.5.7) [51] reducing the data to two dimensions. Before applying UMAP, Hits table values were log2-normalized for scale consistency. To balance local and global structure preservation, the number of neighbors was set to 15 according to the overall number of samples in the smallest group (5 non-infected ferrets in triplicate). The embedding was initialized using the default spectral method, which provides a globally informed starting layout. The random state was fixed to ensure reproducibility. The color scheme used in the UMAP visualization was the same as in the clustermap.

### Motif reduction

Two independent methods were used to focus on the most relevant motifs:

#### (i) Pruning the column dendrogram

As described earlier, the column dendrogram was calculated using the UPGMA algorithm. To define distinct clusters representing individual motifs, the dendrogram was pruned by applying a threshold distance of 21 using the fcluster function from the scipy.cluster.hierarchy module (SciPy version 1.14.1) [46].

#### (ii) Principal Component Analysis (PCA)

Dimensionality reduction of the Hits table, consisting of M motifs, was performed using PCA. Prior to applying PCA, the table values were log2-normalized to standardize the range of quantities. PCA was implemented using the Scikit-learn package (version 1.5.2) [48], reducing the data to two principal components. The color scheme used in the PCA visualization was the same as in the clustermap.

## Results

### Initial analysis of infected vs. non-infected ferret sera

To evaluate *Motifier*’s feasibility for characterizing polyclonal sera, naïve ferrets and ferrets infected with one of several influenza A H3N2 virus strains were used as a model. Serum samples from 38 ferrets were divided into training and test sets. The test set included infected ferrets from two groups: homologous—infected with the same strain as in the training set—and heterologous— infected with strains other than those used in the training set (see Materials and Methods, Table A). Each serum was screened in triplicate against the TAU combinatorial phage-display random peptide library, yielding high-complexity peptide datasets.

For this study, the re-implemented *Motifier* pipeline was applied to the training set, which comprised sera from 5 non-infected ferrets and 9 infected ferrets. The infected animals were divided into three groups of three ferrets, each infected with a distinct HA variant (Kenya, Miyazaki, or Ethiopia). Following the initial quality control and normalization module, a total of 113,572,109 valid reads were retained for downstream analyses. Motif inference was carried out by independently clustering peptides from each barcode (three barcodes per ferret) of the infected ferrets, followed by the merging of similar clusters across individuals. This procedure resulted in the selection of the top 400 clusters further developed into motifs. Collectively, these motifs comprised 30,236 unique peptides with a total abundance of approximately 12 million RPM, corresponding to ~45% of all the peptides used for motif inference. Peptides from all 42 barcoded training samples (infected and non-infected ferrets, a total of 14 ferrets × 3 replicates) were then assigned to these motifs, generating the Hits table (motif abundance matrix, see Supplementary Table S3). After log2-transformation, the matrix was used for downstream analyses.

**Figure 1.**
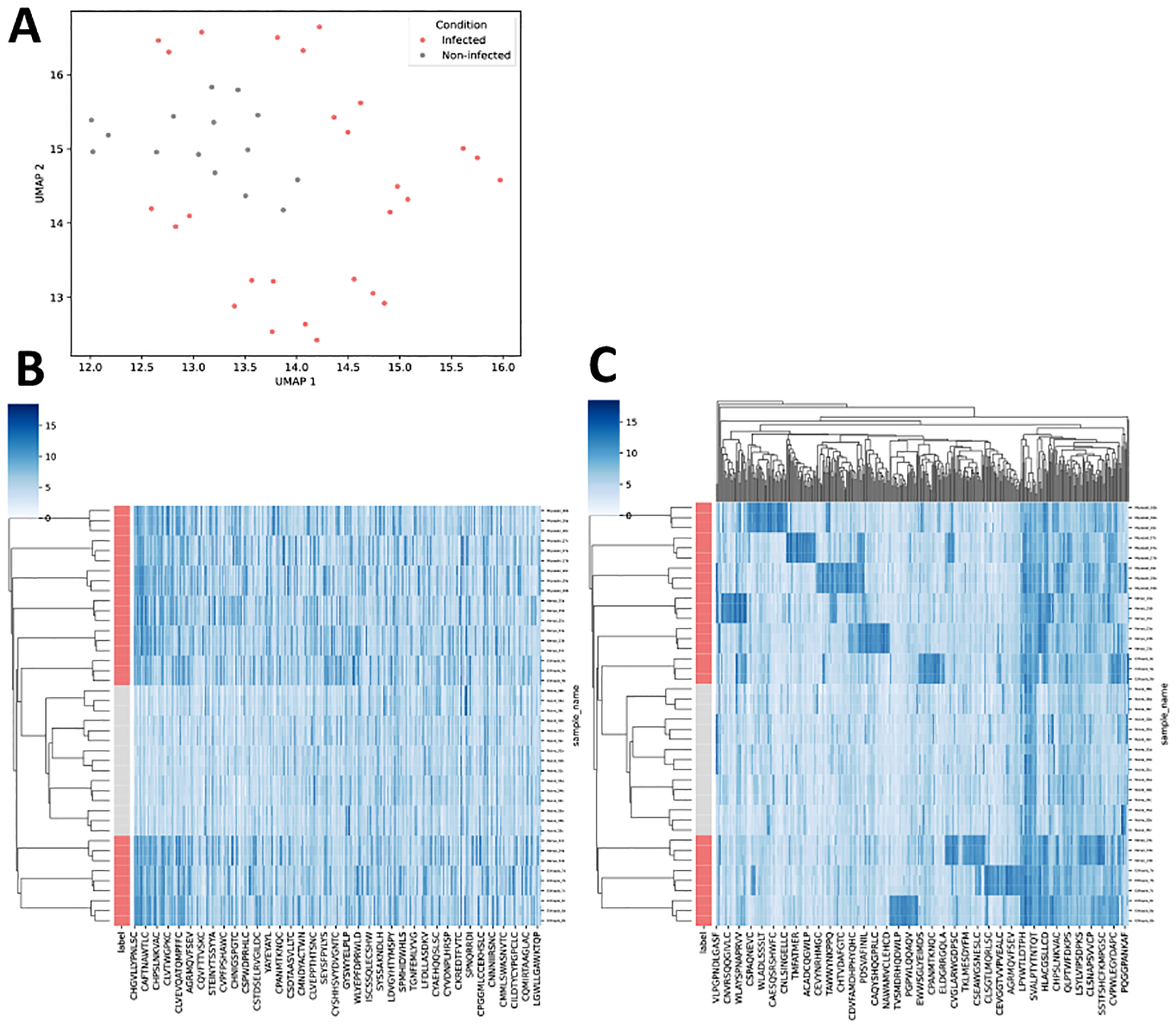
Visualization of 400 motifs derived from infected ferrets across all ferret samples. All panels are based on the log2-transformed Hits table, which represents 400 motifs derived from the 9 infected ferrets and Hits counts from 14 ferrets in the training set, each in triplicate. Samples obtained from infected ferrets are shown in pink, while non-infected ferret samples are shown in gray. A. UMAP results provide a 2-dimensional representation of the samples’ distribution across the motifs. B. Clustermap: The *x*-axis represents the motifs (only 35 representative consensus sequences are displayed). The *y*-axis represents the 14 ferrets in the training set (in triplicate). The dendrogram on the left illustrates the similarity between the ferret samples and the intensity of the color indicates the Hits counts, with higher intensity representing a greater count of Hits from a sample to the corresponding motif. C. This is the same clustermap as in B, with the addition of the columns’ dendrogram illustrating the similarity between the motif patterns across the samples (note, the 35 consensus sequences depicted in panels B and C are not necessarily the same).

Initial dimensionality-reduction, using UMAP, revealed partial separation of the infected *vs*. non-infected ferrets (Figure 1A). Technical replicates grouped tightly for each individual, confirming experimental reproducibility. The group of non-infected samples was more compact, compared with those of the infected ferrets which were more dispersed in the UMAP space. Hierarchical clustering (Figure 1B) confirmed these findings: non-infected ferrets grouped along a central branch, while infected ferrets were distributed across divergent subclusters. Clustermap visualizations further showed consistent intraindividual patterns across replicates.

To further explore this structure, hierarchical clustering was applied to the columns (motifs) of the transformed Hits table (Figure 1C). This analysis revealed that individual infected-ferrets exhibited distinct subsets of motifs. These “personal motifs” appeared as strong, individual- specific signals that generally were not shared among other ferrets. The absence of such personal signatures in the non-infected ferrets may have led to their apparent clustering in the row-wise dendrogram. To further test this, we repeated the motif inference process but inverted the sample sets, i.e., using non-infected ferrets as the motif-generating cohort. The results (Supplementary Figure S4) reflected the initial findings: for the ferrets used to generate the motifs, personal motifs emerged and for other ferrets not used to generate motifs a single branch in the dendrogram was observed (in this case the infected ferrets got clustered). This suggests that the initial separation was indeed influenced by the presence – or more precisely, the absence – of personal motif signals, and not driven by a common immunogenic signature associated with the biological condition of interest.

**Figure 2.**
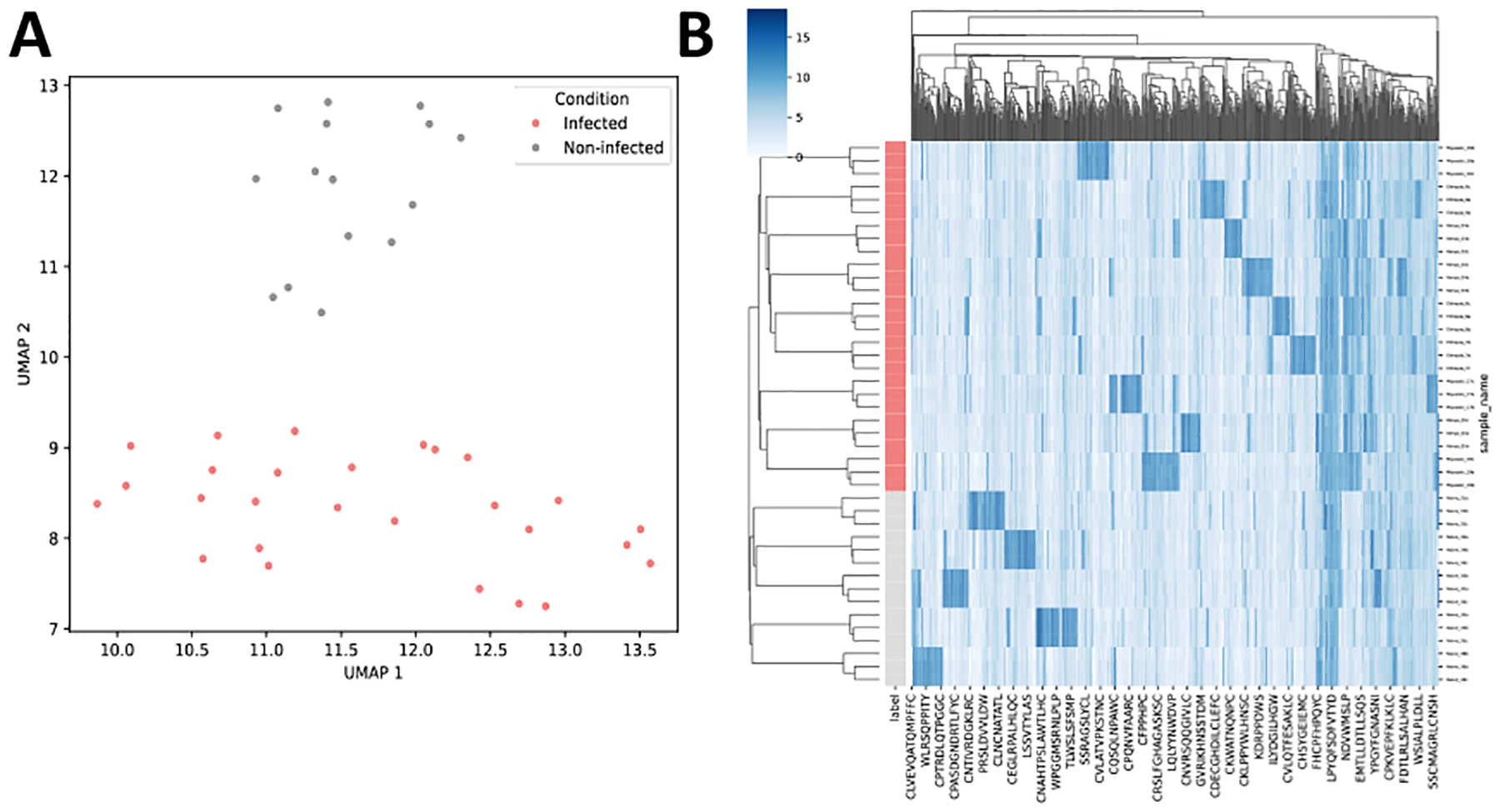
Visualization of 800 motifs derived from infected and non-infected ferrets across all ferret samples. All panels are based on the log2-transformed Hits table, which represents 800 motifs derived from the 14 ferrets in the training set, both infected and non-infected, and Hits counts from the same ferrets. A. UMAP results provide a 2-dimensional representation of the samples’ distribution across the motifs. B. Clustermap results provide a clustered heatmap.

To mitigate the effect of sample separation driven by personal motif signals, we performed a new round of motif inference using the complete set of training samples. Specifically, peptides from both infected and non-infected ferrets were included in the clustering process, generating 800 motifs. As a result, all individual ferrets contributed to motif construction and consequently each exhibited personal motifs. When dimensionality-reduction via UMAP was applied to the updated transformed Hits table (Figure 2A), a clear separation between infected and non-infected samples emerged. Hierarchical clustering of the same data (Figure 2B) similarly produced two distinct branches corresponding to the biological conditions. These results suggest the presence of an underlying shared immune signal among infected ferrets. By deriving personal motifs for all ferrets, we were able to minimize the influence of personal signals as drivers of separation, which allowed the way to identify common signals potentially indicative of infection.

### Focusing on signals common to the infected ferrets

To refine the motif set and reduce noise from individual-specific patterns, we focused on identifying features shared among infected ferrets. A major source of variability was personal motifs – motifs highly enriched in one ferret but absent in the rest.

**Figure 3.**
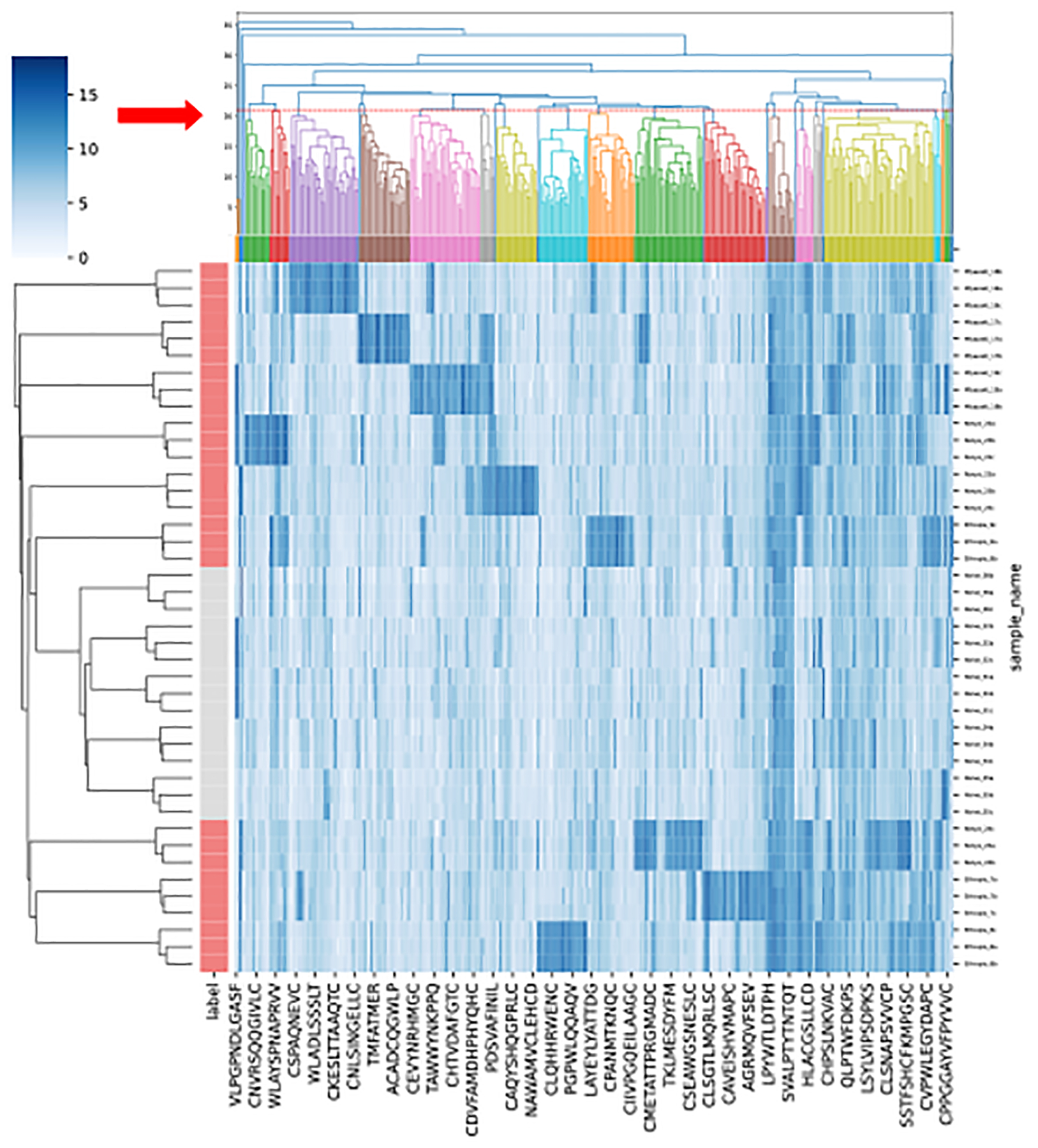
Clustermap showing the 31 color-coded clusters. This clustermap (same as that shown in Figure 1C) is based on the 400 motifs selected to discriminate between infected and non-infected ferrets. Clusters were defined using a dendrogram pruned at a threshold of 21 (arrow and dotted line), with each color representing a distinct cluster. The scale is logarithmic.

We applied two filtering strategies to address this. The first was “dendrogram pruning” based on motif behavior. In the column-wise dendrogram from Figure 1C (400 motifs from the infected ferrets), each ferret formed a distinct subtree, consistent with personal signatures. Using a distance threshold of 21, we clustered the motifs into 31 groups (Figure 3), then manually inspected cluster-specific clustermaps (Supplementary Folder S5). Clusters that exhibited two branches – one corresponding to a single ferret and the other to all remaining samples – were defined as personal clusters, and the motifs comprising those clusters were excluded from further analyses. After filtering, 133 motifs remained for downstream analysis.

**Figure 4.**
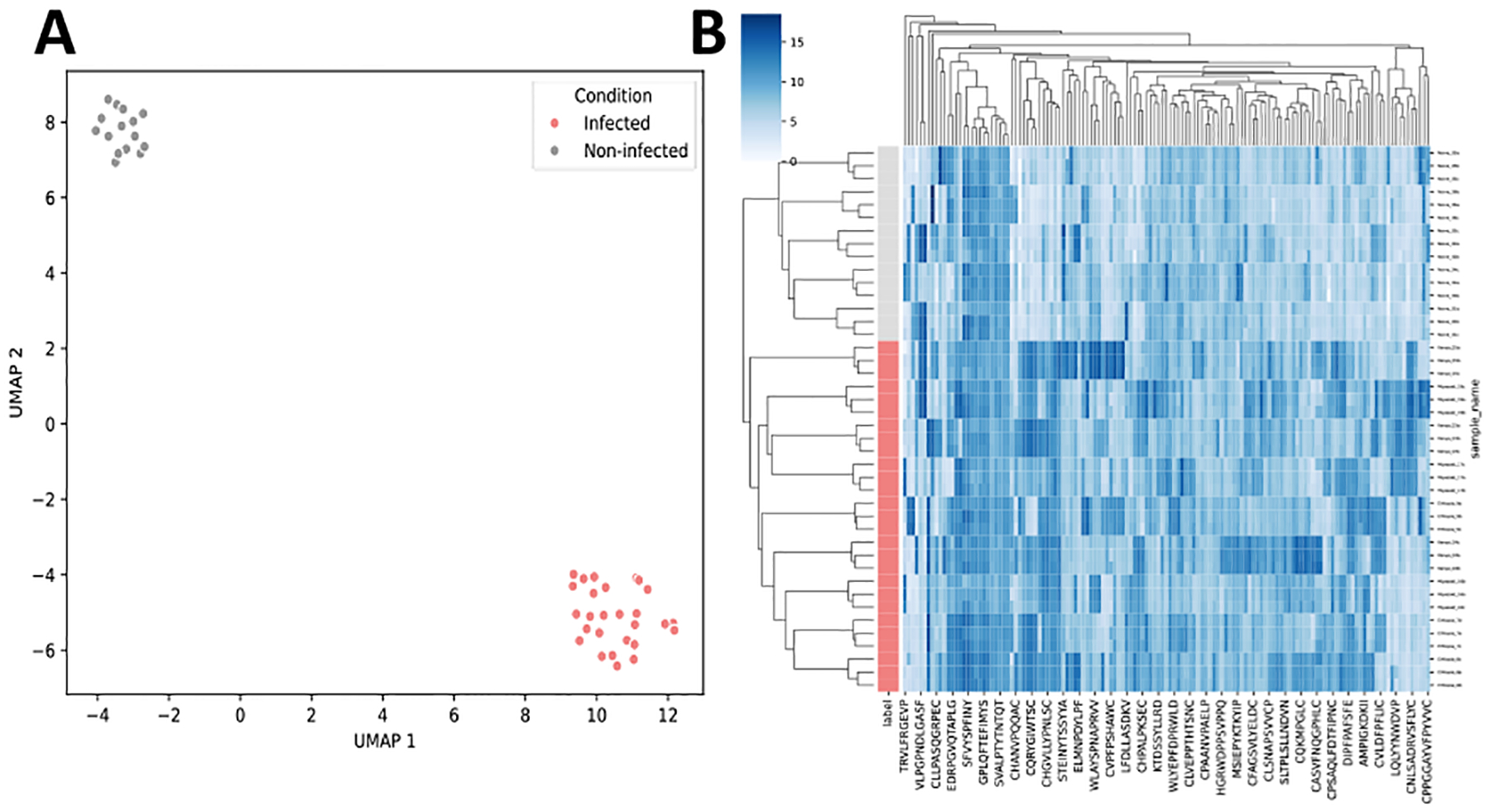
Visualization of 133 motifs derived from infected ferrets across all ferret samples. Both panels are based on the log2-transformed Hits table, which represents the 133 motifs after removing personal motifs. The motifs were derived from the 9 ferrets in the infected biological condition and Hits counts from 14 ferrets in the training set, each in triplicate. A. UMAP results provide a 2-dimensional representation of the samples’ distribution across the motifs. B. Clustermap results provide a clustered heatmap.

As shown in Figure 4A, applying UMAP to the filtered motif-set resulted in a markedly improved separation between infected and non-infected samples. In Figure 4B, replicates from each individual consistently clustered together, and two distinct branches corresponding to the biological conditions were observed.

**Figure 5.**
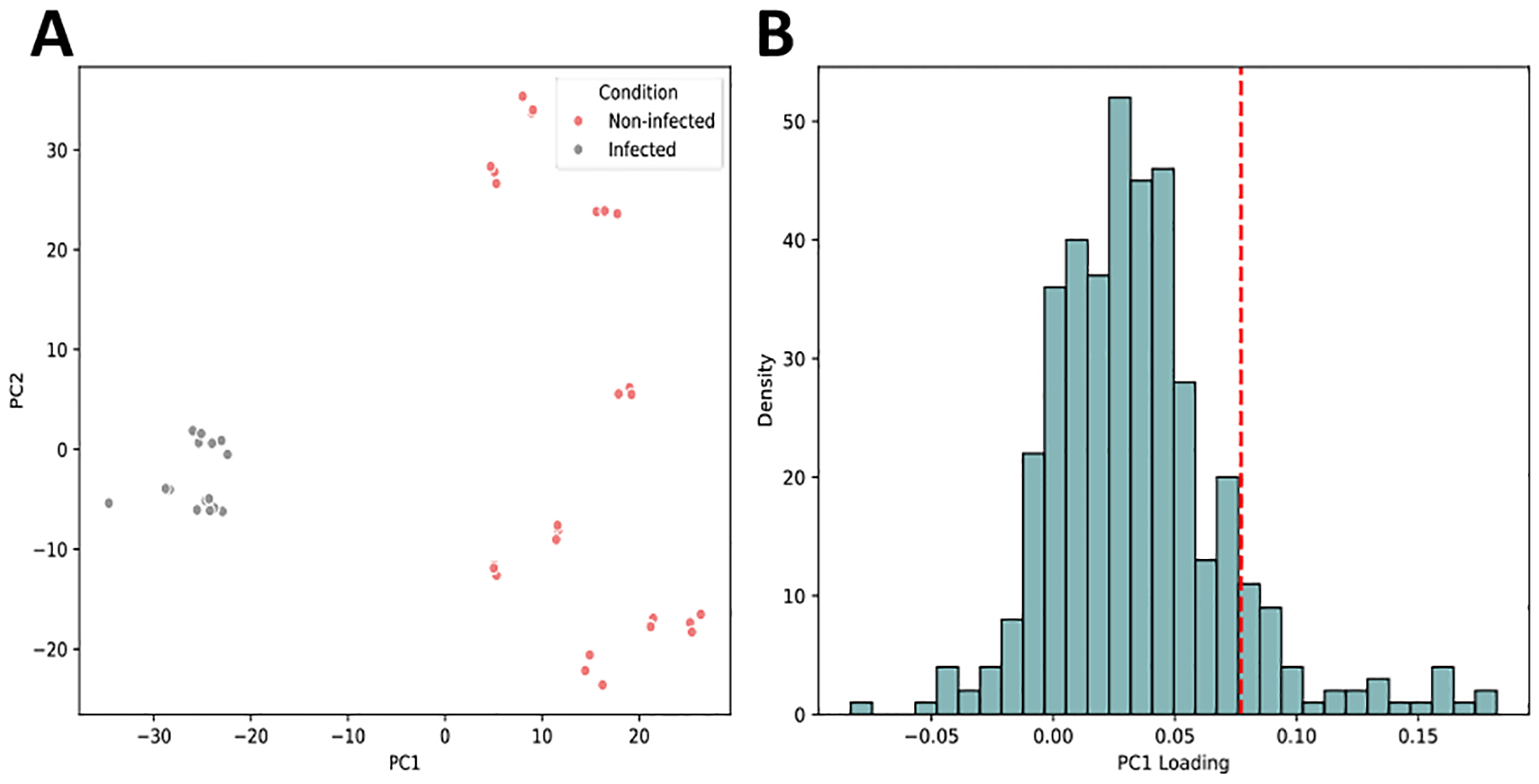
PCA analysis of the 400 motifs derived from the infected ferrets across all ferret samples. **A**. PCA projection based on 400 motifs across 14 ferrets (infected and non-infected) in the training set, each in triplicate, after log2-transformation. Dimensionality was reduced to two principal components, with PC1 showing a clear separation between infected and non-infected ferrets. **B**. PCA loadings plot, illustrating the contribution of individual motifs to PC1. The loading value represents each motif’s influence on the separation, where higher absolute values indicate a stronger impact. Motifs with positive loadings on PC1 were associated with the infected biological condition, while motifs with negative loadings correlated with the non-infected condition. The dashed red line represents the threshold (0.0771), used to select motifs with higher loadings for the next analysis.

**Figure 6.**
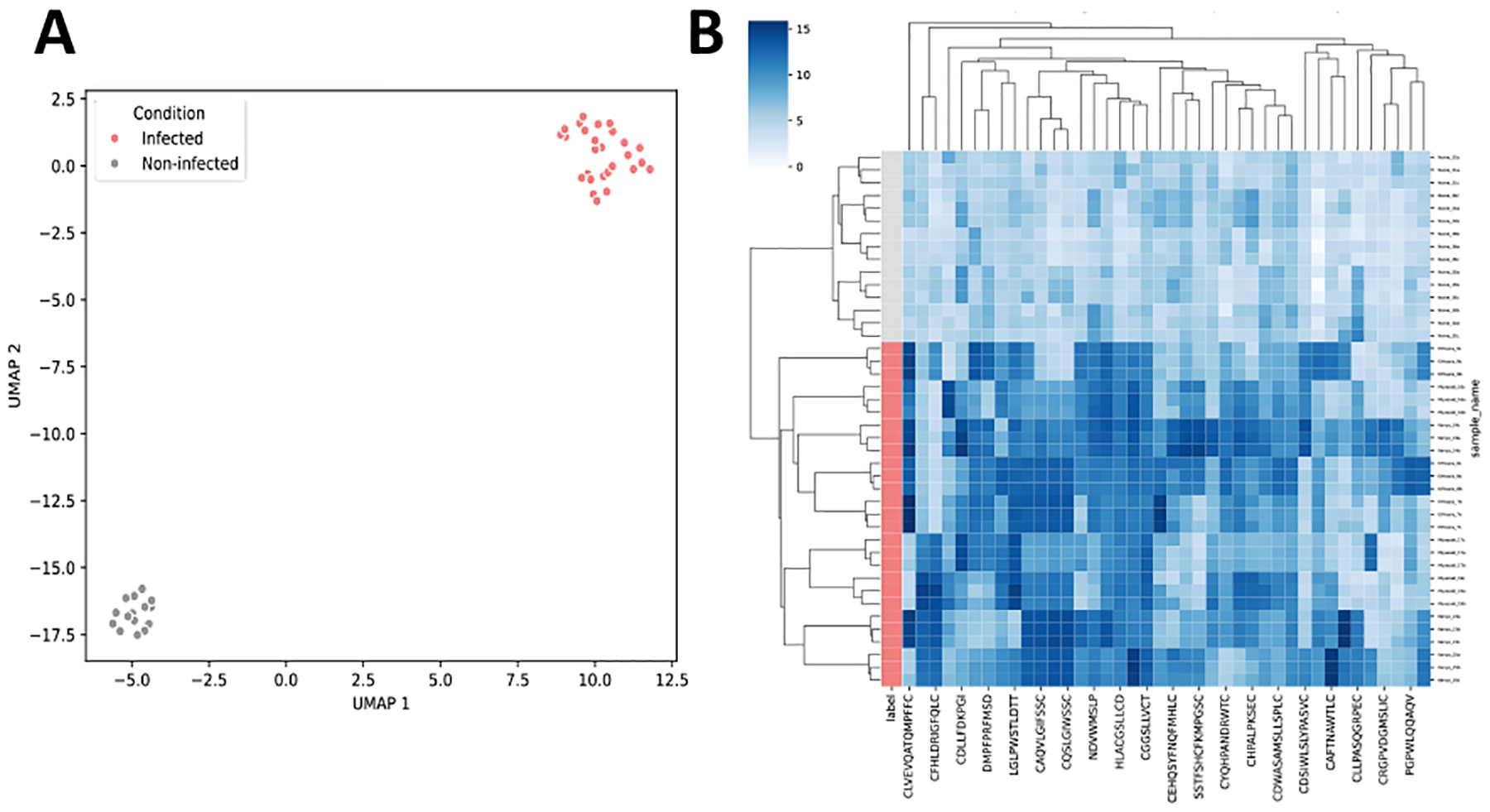
Visualization of the 40 motifs derived from infected ferrets across all ferret samples. Both panels are based on the log2-transformed Hits table, which represents the 40 most positive loadings in the PCA. The motifs were derived from the 9 ferrets in the infected biological condition and Hits counts from 14 ferrets in the training set. Infected ferret samples are shown in pink, while non-infected ferret samples are shown in gray. **A.** UMAP results provide a 2-dimensional representation of the samples’ distribution across the motifs. **B**. Clustermap results provide a clustered heatmap.

Whereas the “pruning” filtering approach focused on removing individual-specific motifs, we next applied an alternative strategy aimed at directly identifying motifs associated with the infection condition. To this end, we employed PCA to determine whether variation in motif abundance could distinguish between infected and non-infected ferrets. PCA was performed on the log2-transformed Hits table containing 400 motifs across all training samples, reducing dimensionality to two principal components (PCs). PCA visualization (Figure 5A) revealed a clear separation between conditions along PC1. Each motif contributed to PC1 with a specific loading value, reflecting its influence on the observed separation. Positive PC1 loadings were associated with infected samples, while negative loadings aligned with non-infected samples. To identify motifs most strongly associated with infection, we focused on those with high positive PC1 loadings. Specifically, we selected motifs above the 90th percentile of the loading distribution (threshold = 0.0771; indicated by a dashed red line in Figure 5B). These motifs were considered the most relevant features for distinguishing the infected condition. Out of the 400 motifs, the forty exceeding the threshold, were selected as the top contributors to PC1. These motifs were evaluated as candidate discriminative features (the full list of consensus sequences of the motifs is provided in Supplementary Table S6). Despite representing a relatively small subset, these 40 motifs collectively accounted for 3333 unique peptides (~2M RPM), comprising approximately 7% of the peptide pool used for motif inference. UMAP projection based on this reduced motif set (Figure 6A) revealed a clear separation between infected and non-infected ferrets. Clustermap visualization (Figure 6B) further supported this distinction, maintaining consistent grouping of technical replicates and producing two major branches corresponding to the biological conditions. Notably, the selected motifs appeared to represent a shared immunological signal among multiple infected ferrets, while being largely absent from non-infected samples.

### Predictive evaluation using random forest

To assess whether the 40 identified motifs could serve as predictive features, we implemented a RF classifier. A total of 100 models were trained using different combinations of six hyperparameters. The final model was selected based on its performance, achieving a zero error rate on the training data.

Model generalization was evaluated on a test set that included three non-infected ferrets (in triplicate, i.e. 9 samples) versus two subgroups of infected ferrets: homologous and heterologous (see Table A). The homologous group included 10 infected ferrets exposed to the same three H3N2 strains used in the training set (Kenya, Miyazaki, and Ethiopia) screened in triplicate (one Kenya-infected ferret is missing a replicate due to low sequencing depth, thus yielding a total of 29 infected samples). The heterologous group included 11 ferrets infected with three different H3N2 strains not represented in the training set (Osorno, Missouri, and Singapore) screened in triplicate, for a total of 33 samples.

**Figure 7.**
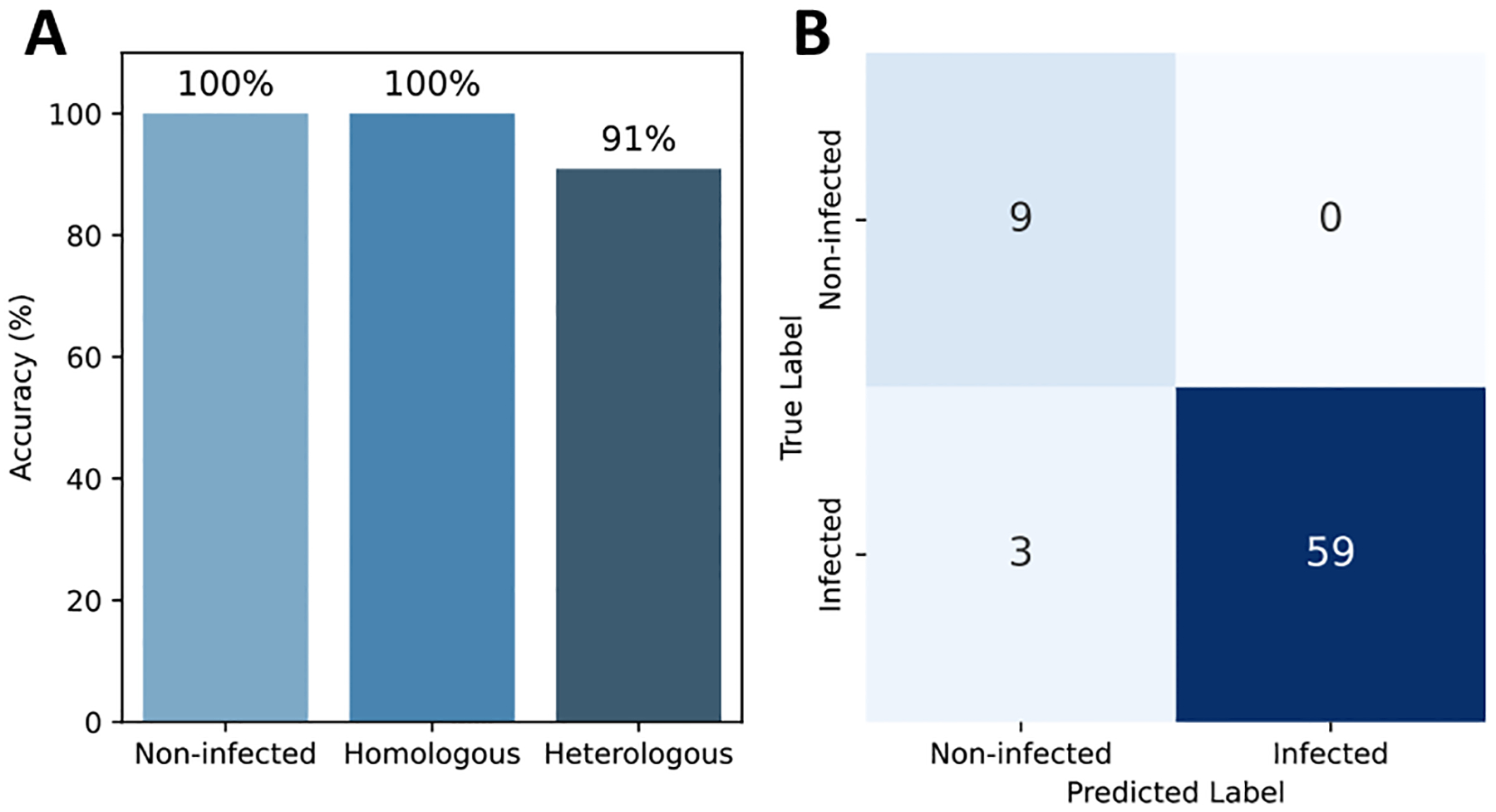
RF performance. **A**. Classification accuracy for non-infected, homologously infected, and heterologously infected ferret samples. The model achieved 100% accuracy for non-infected and homologous infections, and 91% accuracy for heterologous infections. **B.** Confusion matrix summarizing prediction outcomes across all test samples. The classifier correctly identified all non-infected and nearly all infected samples, with only three heterologously infected samples (triplicates from a single ferret) misclassified as non-infected.

Prediction was performed by reapplying the 40 selected PCA-filtered motifs to the test set peptides, generating a new Hits table as model input. In the test set, both the non-infected and homologous samples achieved 100% accuracy, correctly classifying all infected and non-infected ferrets. In the heterologous group, three samples from a single ferret (Osorno_2) were misclassified, yielding an accuracy of approximately 91% for that subgroup. Overall, the model achieved ~96% accuracy (Figure 7). Importantly, it maintained perfect specificity and high sensitivity, indicating that the identified motifs robustly capture infection-associated features across strains, including previously unseen variants.

These findings demonstrate that the combination of 40 motifs, selected based on PC1 loadings, effectively distinguished infected ferrets from non-infected ones. Moreover, this distinction remained robust not only for ferrets included in model training but also for unseen ferrets infected with different H3N2 strains that were not present in the training set. This result highlights the biological relevance of the selected motifs and their potential as generalizable predictive markers for infection status.

## Discussion

The primary objective of this study was to determine whether affinity-selected peptides corresponding to serum samples could serve as markers for identifying shared immunological signals that distinguish between different biological conditions. To address this question, we applied DP combined with the *Motifier* computational framework to profile antibody responses in polyclonal sera from ferrets infected with the H3N2 subtypes of influenza A virus.

While *Motifier* was originally developed for profiling of mAbs, in this study it was adapted to the more complex setting of polyclonal serum. After applying *Motifier* to several DP experiments, we re-implemented the pipeline to achieve faster execution and more streamlined analysis. This re-implementation precisely reproduced the results of the original publication, ensuring full methodological reproducibility before extending its application to the current study.

Unlike mAbs, which recognize a single epitope, polyclonal serum contains a diverse and dynamically evolving repertoire of antibodies targeting multiple epitopes derived from numerous antigens – a feature that has historically posed challenges [52]. This repertoire is shaped by the individual’s unique history of antigen exposure, meaning that the specific signal associated with a particular biological condition is embedded within a complex background of unrelated binding events. Applying the re-implemented *Motifier* to polyclonal serum enabled the identification of motifs associated with infection-induced immune responses. When combined in a RF classifier, these motifs also demonstrated predictive potential in sera from ferrets infected with heterologous influenza subtypes, highlighting their value as generalized diagnostic markers.

According to established immunological principles, adaptive immunity is initiated upon first pathogen exposure, leading to a peak of pathogen-specific antibodies shortly after infection or vaccination, followed by a decline during resolution [53,54]. Consistent with this, we initially expected to observe a dominant peak of pathogen-specific antibodies in infected ferrets. However, our analysis revealed motifs that were markedly stronger within individual animals, termed “personal motifs”, which appeared unique and out-weighed shared pathogen-driven signals. Similar observations were reported in a recent study using liquid chromatography-mass spectrometry (LC-MS) to analyze donors after COVID-19 vaccination [55]. That study demonstrated that over a year and after three vaccine doses, the antibody repertoire remained largely personal and remarkably stable. This finding supports our observation of dominant individual-specific antibody motifs despite repeated antigen exposures and highlights the complexity of the immune response shaped by personal history and background, which can obscure common signals associated with infection.

To overcome the background signal, we applied two computational filtering approaches: first, manual pruning, and second, statistical filtering using PCA loadings. These approaches improved the identification of common, infection-specific motifs embedded within the background noise of personal repertoires.

Following the second approach, we developed a RF classifier based on 40 motifs, which effectively distinguished infected from non-infected serum samples with high accuracy. In view of the fact that this was found efficient for both test-sets, homologous and heterologous, the selected motifs are regarded as generalized diagnostic markers. Such cross-reactive markers are of particular interest because they may detect broader immune patterns that transcend strain-specific or personal differences, making them promising candidates for the development of broadly applicable serological assays or universal vaccines.

Feature importance analysis for the RF using the Gini index [49,50] revealed that no single motif dominated classification; rather, the model’s performance relied on the combined contribution of multiple motifs, underscoring the value of their combinatorial effect. These findings align with the concept of “combinatorial diagnostics”, where the combination of individually weak markers provides stronger diagnostic power than any single feature alone [56,57]. This approach is especially relevant in immunology, where immune responses are typically characterized by diverse and subtle motifs rather than a single dominant epitope. By leveraging combinatorial signatures, our results support more robust and nuanced serological classification strategies for infectious diseases.

## Conclusion

This study demonstrates that DP using phage-display random peptide libraries and *Motifier* analysis can extract motifs that characterize the antibody repertoire in polyclonal serum. By removing dominant personal immune signatures and focusing on motifs shared among ferrets representing a given biological condition, we identified a set of motifs likely generated in response to that condition. These motifs proved to be cross-reactive, and their combinatorial integration enabled the construction of a model that achieved high predictive performance on unseen data involving different influenza H3N2 subtypes. By distinguishing between personal, condition-specific and cross-reactive signals, we can refine and generalize biomarkers applicable across individuals and potentially across pathogen strains. Owing to their robustness and cross-reactivity, these motifs hold strong potential as the basis for broadly applicable serological diagnostics for infectious diseases.

## Supplementary Material

wird description

zip folder

## Figures and Tables

**Table A:** Experimental design and distribution of ferrets across study groups.

Group & subtype		Total	Training set	Test set
Non-infected	Naïve (PBS)	8	5	3
Infected	A/KENYA/105/2017 (Kenya)	7	3	4
	A/MIYAZAKI/89/2017 (Miyazaki)	7	3	4
	A/ETHIOPIA/1877/2017 (Ethiopia)	5	3	2
	A/OSORNO/60580/2017 (Osorno)	4		4
	A/MISSOURI/37/2017 (Missouri)	3		3
	A/SINGAPORE/INFIMH160019/2016 (Singapore)	4		4
Total		38	14	24

The table summarizes the number of ferrets in each experimental set, specifying infection status (infected *vs*. non-infected) and the H3N2 strains used for infection. The training set includes ferrets intranasally infected with one of three distinct strains (Kenya, Miyazaki, Ethiopia). The test set comprised 3 non-infected ferrets, ferrets infected with the same strain as the training set (“Homologous”) and ferrets infected with a different strain (“Heterologous”).

## Data Availability

The raw sequencing data produced and used in the present study are deposited in NCBI (accession SAMN55342667).
