# Supplementary material for: Serum-antibody Profiling of H3N2-infected Ferrets Using a Combinatorial Phage-display Random Peptide Library
Source: J Mol Biol. Author manuscript; Available in PMC 2026 Jul 15. (PMC13348039; doi:10.1016/j.jmb.2026.169816)
Supplement: wird description [file NIHMS2181427-supplement-wird_description.docx]

**Supplementary**

**File S1: FASTA file with “irrelevant” peptides.**This file contains peptides identified as “irrelevant” across repeated HTS analyses. These peptides showed consistent enrichment independent of serum specificity and were therefore excluded from all downstream analyses.

**Table S2: Summary of Module 1 – Quality Control from the DP experiment.**
The table contains seven columns: barcode, sample name, set, peptide counts before filtration, peptide counts after filtration, unique peptides after filtration and 1 RPM score. Quality control filtering criteria: barcode accuracy; constant-region mismatch threshold (≤1); NNK-consistent random insertion; and insert lengths of 4–12 amino acids, with and without flanking cysteine.

**Table S3: Infected motifs Hits table.**Columns represent the 400 motifs inferred from the nine infected ferrets, and rows correspond to the serum samples of the 14 ferrets in the training set, each analyzed in triplicate. Each cell reports the abundance (in RPM) of peptides from a given sample assigned to the corresponding motif.

**Figure S4: Visualization of 400 motifs derived from non-infected ferrets across all ferret samples.**
All panels are based on the log2-transformed Hits table, which represents 400 motifs derived from the 5 non-infected ferrets and Hits counts from 14 ferrets in the training set, each in triplicate. Note: non-infected ferret samples are shown in pink, while infected ferret samples are shown in gray. **A.** UMAP results provide a 2-dimensional representation of the sample's distribution across the motifs. **B.** Clustermap results provide a clustered heatmap.

**Folder S5:** **Clustermaps for each branch remaining after manual pruning.**Each map displays the hierarchical clustering results of the motifs within that branch. Clusters showing two distinct branches - one comprising peptides from a single ferret and the other from all remaining samples - were classified as "personal clusters" and were removed.

**Table S6**:
The list of 40 selected motifs was used as features in the RF model.
