# Supplementary figures and images for: Serum-antibody Profiling of H3N2-infected Ferrets Using a Combinatorial Phage-display Random Peptide Library
Source: J Mol Biol. Author manuscript; Available in PMC 2026 Jul 15. (PMC13348039; doi:10.1016/j.jmb.2026.169816)

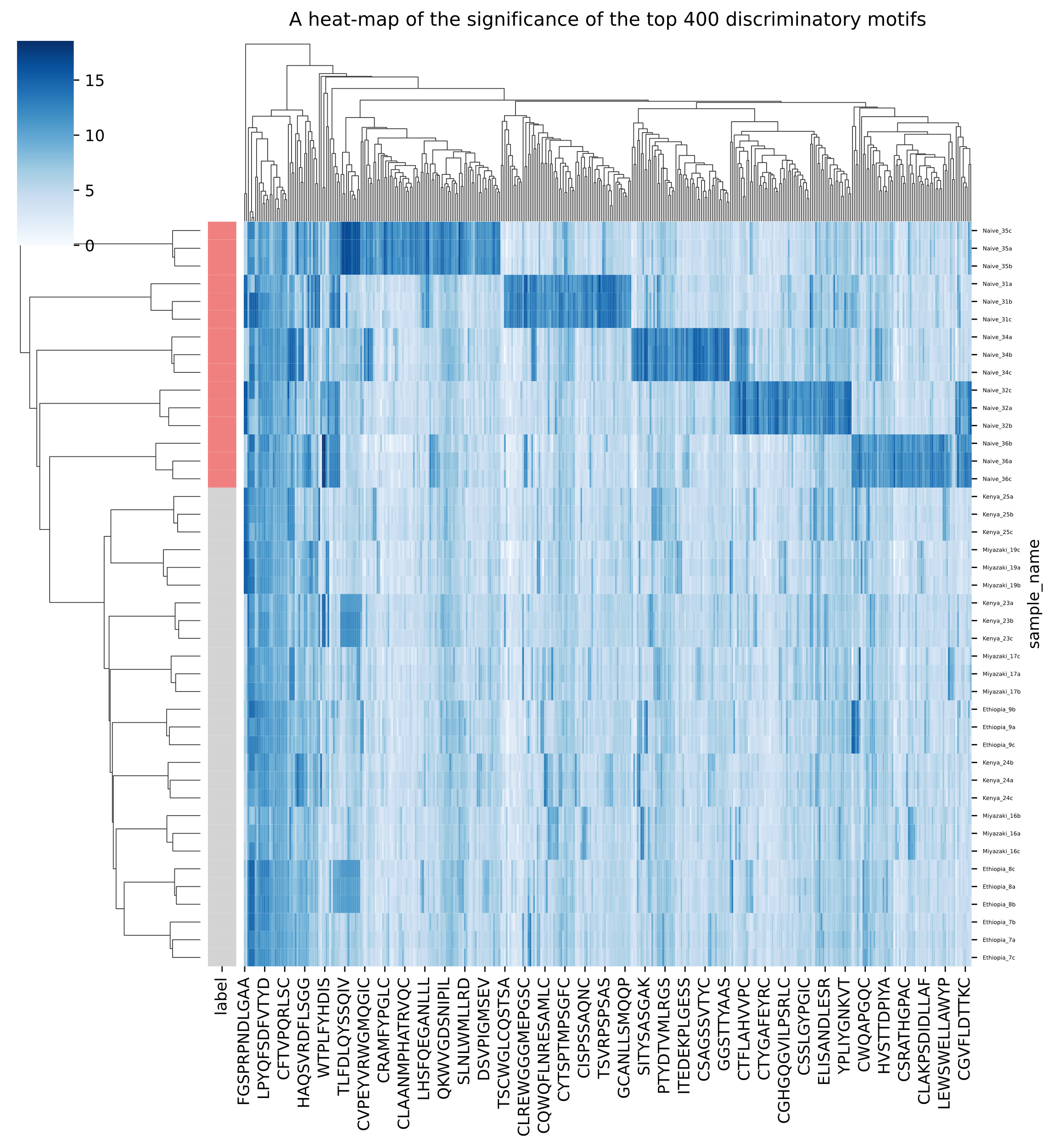


**A**


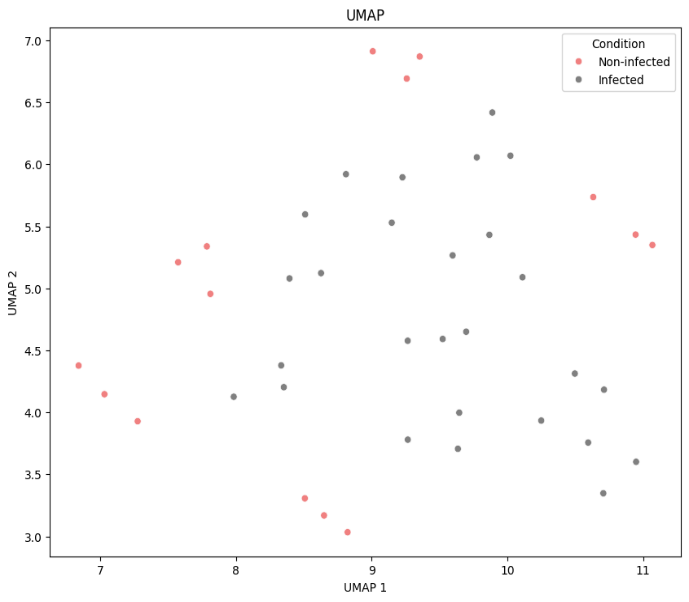


**B**

**A**

Supplement: zip folder [file NIHMS2181427-supplement-zip_folder.zip › Figure S4.docx]
